# Detection of Pathogenic *Escherichia coli* in Samples Collected at an Abattoir in Zaria, Nigeria and at Different Points in the Surrounding Environment

**DOI:** 10.3390/ijerph120100679

**Published:** 2015-01-13

**Authors:** Lawan Mohammed Kabiru, Mohammed Bello, Junaid Kabir, Laura Grande, Stefano Morabito

**Affiliations:** 1 Department of Veterinary Public Health and Preventive Medicine, Ahmadu Bello University, P.M.B. 1096, Zaria 2222, Nigeria; E-Mails: kaboskylawal@yahoo.com (L.M.K.); mbrobah@yahoo.com (M.B.); jkabng@yahoo.com (J.K.); 2 EU Reference Laboratory for *E. coli*, Veterinary Public Health and Food Safety Department, Istituto Superiore di Sanita, Viale Regina Elena 299, Rome 00161, Italy; E-Mail: stefano.morabito@iss.it

**Keywords:** Shiga toxin-producing *E. coli*, slaughterhouse, environmental contamination

## Abstract

Pathogenic *Escherichia coli* can be released with the wastes coming from slaughterhouses into the environment, where they can persist. We investigated the presence of diarrheagenic *E. coli* in specimens taken at an abattoir located in the Zaria region, Nigeria, in samples of water from the river Koreye, where the effluent from the abattoir spills in, and vegetable specimens taken at a nearby farm. All the isolated *E. coli* were assayed for the production of Shiga toxins (Stx) by using the Ridascreen verotoxin Immunoassay and by PCR amplification of genes associated with the diarrheagenic *E. coli*. Three strains from the rectal content of two slaughtered animals and a cabbage were positive for the presence of the Stx-coding genes. Additionally we have isolated one Enteroaggregative *E. coli* (EAggEC) from the abattoir effluent and two Subtilase-producing *E. coli* from the slaughterhouse’s effluent and a sample of carrots. Our results provide evidence that pathogenic *E. coli* can contaminate the environment as a result of the discharge into the environment of untreated abattoir effluent, representing a reservoir for STEC and other diarrheagenic *E. coli* favouring their spread to crops.

## 1. Introduction

*Escherichia coli* is a ubiquitous bacterial species commensal of humans and warm blooded animals. Nevertheless some strains have evolved the capability to cause both intestinal and extraintestinal illnesses [1,2]. The different pathogenic *E. coli* are characterized by particular subsets of genes associated with the virulence, identifying distinct groups or pathogroups. Many amongst the enteric infections caused by *E. coli* are transmitted by inter-human contacts such as those caused by Entero-invasive *E. coli* (EIEC), Enteropathogenic *E. coli* (EPEC) or Enteroaggregative *E. coli* (EAggEC) [3,4], while those ascribed to Enterotoxigenic *E. coli* (ETEC) or Shiga toxin-producing *E. coli* (STEC), are primarily transmitted to humans through the consumption of contaminated water or food [5,6]. STEC cause a wide range of human diseases, including mild-to-severe diarrhoea to haemorrhagic colitis (HC) and the life-threatening haemolytic uremic syndrome (HUS) [6] and are characterised by the production of potent cytotoxins, the Shiga toxins (Stx), whose coding genes are conveyed by temperate bacteriophages [7]. Pathogenic *E. coli* with an inter-human circulation represent a leading cause of diarrhoea, often with high mortality rates, in developing countries [3]. On the other hand, STEC have gained increasing global concern as food-borne pathogens worldwide [6,8,9] and are the only diarrheagenic *E. coli* pathogroup with an ascertained zoonotic origin, with ruminants being regarded as the main animal reservoir [10,11]. It has been hypothesized that the typical STEC isolated from cases of HC and HUS, also termed Enterohaemorrhagic *E. coli* (EHEC) [12], evolved from EPEC or EPEC-like strains following an event of *stx*-phage acquisition [13,14]. In fact, EHEC share with EPEC the capability to colonize the intestinal mucosa with a mechanism known as Attaching and Effacing, mediated by the presence of a pathogenicity island termed the locus for enterocyte effacement (LEE) [15]. Similarly to EHEC, the Enteroaggregative Haemorrhagic *E. coli* (EAHEC) seem to have emerged following an event of *stx*-phage acquisition by an EAggEC [16]. EAHEC broke the scene in 2011 when a large outbreak of infections with a STEC O104:H4 hit Germany and France [17,18]. However, the isolation of EAHEC strains from sporadic cases and outbreaks of HUS have been reported since the beginning of the 90 s [19] and up to the present [20].

Both EHEC and EAHEC may have arisen following an event of *stx*-phage acquisition occurring in the intestine of a mammalian host or the environment [21,22,23]. Evidence has been provided showing that the EAHEC O104:H4 that caused the German outbreak in 2011 could have evolved from an EAggEC O104:H4 following the uptake of a *stx2*-phage of bovine origin [16]. In particular geographic regions, such as in some developing countries, infections with diarrheagenic *E. coli* transmitted by inter-human contacts, such as EAggEC, are endemic [3,4]. Additionally, the treatment of human sewages is often ineffective or even absent, causing the wide dispersion of these pathogens in the environment where they may come into contact with STEC or free *stx*-phages originating from ruminants’ excreta. In such a setting, the effluent from slaughterhouses may represent an additional source of environmental contamination with STEC and the related *stx*-phages.

We investigated the presence of pathogenic *E. coli*, particularly STEC, in specimens taken at a large abattoir, in a water stream receiving the effluent from the slaughterhouse and in a farm producing vegetables that used water from the river to irrigate crops located in the Zaria region, Nigeria. We found that such an ecosystem is an environmental source of STEC and other human-borne pathogenic *E. coli* for vegetables’ contamination and represents a proper milieu for the emergence of strains with shuffled virulence determinants.

## 2. Results

### 2.1. E. coli Isolation and Characterization Using the Ridascreen Verotoxin Immuno Assay

All the colonies isolated from the samples and confirmed as *E. coli* were assayed for the capability to produce Shiga toxins using the Ridascreen Verotoxin Enzyme Immunoassay (EIA) (R-Biopharm Darmstadt, Germany). The results are reported in Table 1. In detail, 183 out of the 200 faecal samples yielded colonies resembling *E. coli* on EMB. Biochemical confirmation of single colonies (one colony per sample) returned positive results for 152 of them. The isolation procedure from the vegetables produced 204 *E. coli* confirmed colonies. Twenty-five and 12 colonies from faecal and vegetables samples, respectively, were positive to the EIA (Table 2). As far as the effluent samples were concerned, five of the 135 confirmed *E. coli* were also positive to the EIA. Finally only one out of the 31 *E. coli* colonies isolated from the water samples gave positive results when subjected to the EIA.

**Table 1:** Results of the *E. coli* isolation on EMB agar and Ridascreen Verotoxin EIA screening.

Sampling Point	No. of Samples		No. of Colonies	
	Collected	Producing Typical Colonies on EMB (%)	Biochemically Confirmed as *E. coli* (%)	Positive to EIA (%)
Faecal	200	183 (91.5)	152 (76)	25 (12.5)
E-1	60	53 (88.3)	48 (80)	4 (6.7)
E-2	60	48 (80)	42 (70)	1(1.7)
E-3	60	52 (86.7)	45 (75)	0 (0)
Carrot	100	79 (79)	68 (68)	6 (6)
Cabbage	100	75 (75)	66 (66)	4 (4)
Lettuce	100	78 (78)	70 (70)	2 (2)
S-1	20	17 (85)	15 (75)	1 (5)
S-2	20	10 (50)	7 (35)	0 (0)
S-3	20	12 (60)	9 (45)	0 (0)

### 2.2. Characterization of the Ridascreen Verotoxin Immunoassay-Positive Colonies by Vero Cell Assay

Of the 43 isolates positive to the Ridascreen Verotoxin EIA only 37 could undergo further characterization. Six strains (four from faecal samples, one from carrot and one from water) could not be recovered after storage in nutrient slant at 4 °C for an eight-months period before being shipped to the European Reference Laboratory for *E. coli* (Rome, Italy).

Vero cell assay (VCA) was used to confirm the production of Shiga toxins. The VCA was carried out using the supernatant of overnight cultures of the 37 remaining strains and revealed that only eight induced a cytopathic effect upon incubation up to 72 h. In particular, two *E. coli* isolated from faecal samples, one from cabbage and one from an effluent sample induced a CPE after 24 h from the inoculum, while four samples, two *E. coli* isolated from cattle faeces, one from carrot and one from cabbage, produced a CPE after 48 h.

### 2.3. Characterisation of the Isolates by PCR Amplification of Virulence Genes

The Ridascreen Verotoxin EIA-positive isolates were subjected to PCR amplification of the genes encoding the Shiga toxins and the intimin-coding *eaeA* gene. All the 29 strains negative in the VCA were also negative in the PCR specific for the *stx*_1_ and *stx*_2_ genes. Three out of the eight strains, which induced a CPE onto Vero cells monolayers gave positive results for the presence of both the *stx*_1_ and *stx*_2_ genes, while none of the 37 isolates produced amplicons when the *eaeA* PCR was carried out (Table 2). All the 37 strains were also subjected to PCR for the detection of genes associated with the other *E. coli* causing intestinal disease such as the EAggEC, EIEC, ETEC and the Subtilase-producing *E. coli*. Such a screening led to the identification of one EAggEC and two Subtilase-producing *E. coli*, while all the samples were negative for the presence of LTh, STh and STp genes of ETEC and for the *ipaH* gene typically associated with EIEC.

**Table 2:** Characterization of the pathogenic *E. coli* strains by PCR, and Vero Cell Assay.

Source	Presence of								
	*VCA*	*stx*_1_	*stx*_2_	*eaeA*	*aggR*	*aaic*	*aat*	*ipaH*	*subAB*
Faecal	+	+	+	-	-	-	-	-	-
Faecal	+	+	+	-	-	-	-	-	-
Faecal	+	-	-	-	-	-	-	-	-
Faecal	+	-	-	-	-	-	-	-	-
Effluent	-	-	-	-	+	-	+	-	-
Effluent	+	-	-	-	-	-	-	-	+
Carrot	+	-	-	-	-	-	-	-	+
Cabbage	+	+	+	-	-	-	-	-	-
Cabbage	+	-	-	-	-	-	-	-	-

## 3. Discussion

*Escherichia coli* is part of the natural microflora of the digestive tract of human and animals but certain strains have evolved the capability to cause a wide range of diseases [1,2]. *E. coli* has a biphasic lifestyle and can effectively survive either in the host or in the environment where it is released with human sewage and animal excreta. Pathogenic *E. coli* with a zoonotic origin, such as STEC, can also be released in the environment with the discharges from slaughterhouses [24]. The abattoir effluents serve as an excellent medium for bacterial multiplication because of its content of faecal matter and blood from slaughtered animal. Waste material is washed off with water from the abattoir operation and, if the effluent is improperly treated for decontamination, it can contaminate the surrounding environment [24]. In the area concerned by the present study no treatment is applied to the abattoir effluent before it is released in the environment and spills into the small river Koreye. The water from the stream in turn is used to irrigate crops grown at a nearby farm.

The main focus of this study was the detection of STEC from specimens taken at different sampling points including the animals slaughtered, the effluent from the abattoir, the water from the river stream and the vegetables grown in the nearby farm in order to verify the possibility that pathogenic *E. coli* from the abattoir could be released in the environment and transmitted to vegetables. We have used the Ridascreen Verotoxin immunoassay kit (R-Biopharm) to screen isolated *E. coli* strains for the production of Shiga toxins (Stx). The choice of the screening approach was due to the lack, in the study area, of the possibility to use the PCR for the detection of virulence genes and was based on previous data published in the literature showing that this commercial method proved as sensitive as the PCR in detecting STEC [25]. Such an approach identified 43 positive specimens collected from all the sampling points, suggesting an extensive environmental contamination with STEC from the abattoir. Unfortunately, only 37 out of the 43 EIA-positive strains survived the long storage before being sent to the European Reference Laboratory for *E. coli* in Rome, Italy, and only eight of these were confirmed as STEC by PCR and Vero cell assay. This finding seems to be in disagreement with how previously published on the performance of the Ridascreen Verotoxin Kit [25,26] but could be explained by possibility that some of the strains produced factors, different from the Stx, cross reacting with the antibodies used in the EIA. Another possibility to explain the discrepancy between the results obtained with the immunoassay and the following characterization of the isolates as STEC may reside in the long storage the strains underwent before being shipped to the European Reference Laboratory for *E. coli* for further analyses. The strains have been kept for eight months as slant agar cultures at 4 °C and might have lost their *stx*-phages during the storage. Accordingly, the SubAB-producing strains identified in this study could derive from STEC that have lost the *stx*-phage. As a matter of fact, the Subtilase cytotoxin has been proposed to be an accessory virulence factor of LEE-negative STEC isolated from human cases of diarrhoea and from animals including cattle and small ruminants [27].

It is interesting to note that one of the three confirmed STEC was isolated from a cabbage sampled in the farm that used the water from the stream to irrigate the crops. The STEC had the same *stx* genes profile of the other two STEC isolated from the animals at the slaughterhouse. Additionally, the two SubAb-positive *E. coli* strains were isolated from the abattoir effluent and from carrots grown at the same farm. These observations suggest that the transfer of pathogenic *E. coli* from the animals to the crops could have occurred through the water contaminated by the effluent from the slaughterhouse. This hypothesis is supported by previous reports describing the presence of *E. coli* O157 in the water of the same river [28] and the identification of bacterial counts above the recommended levels into water bodies in Abuja, Nigeria, due to the release of untreated abattoir wastewater [29].

We have also screened the *E. coli* strains for other known virulence factors of *E. coli* causing intestinal disease in humans. Interestingly, one of the strains isolated from the abattoir effluent, was positive for the presence of *aggR* and *aatA* genes, the genetic determinants of the Enteroaggregative *E. coli* (EAggEC) [30]. According to the literature, EAggEC are highly adapted to humans, suggesting that the human population is their reservoir [31,32]. Additionally, the isolation of EAggEC from animals including cattle and abattoir effluent has been unsuccessfully attempted in different studies [33,34]. In the abattoir visited in the present study, the isolation of an EAggEC is noteworthy and could be the result of contamination deriving from the workers. In fact, the absence of hygiene and standard operating procedures in this operation has been previously reported [35].

Interestingly the isolated EAggEC was positive to the Ridascreen Veroytoxin immunoassay. Given the mentioned possibility that the strains under investigation may have lost the *stx*-phages during the storage, we could have isolated an Enteroaggregative Haemorrhagic *E. coli* (EAHEC). These strains are EAggEC producing Stx [36] that have made their appearance in the 90’s and caused sporadic cases of infection in different countries and at least three epidemics in Europe [20], with one of them being the most severe STEC outbreak ever reported [17].

## 4. Materials and Methods

### 4.1. Study Area

**Figure 1:**
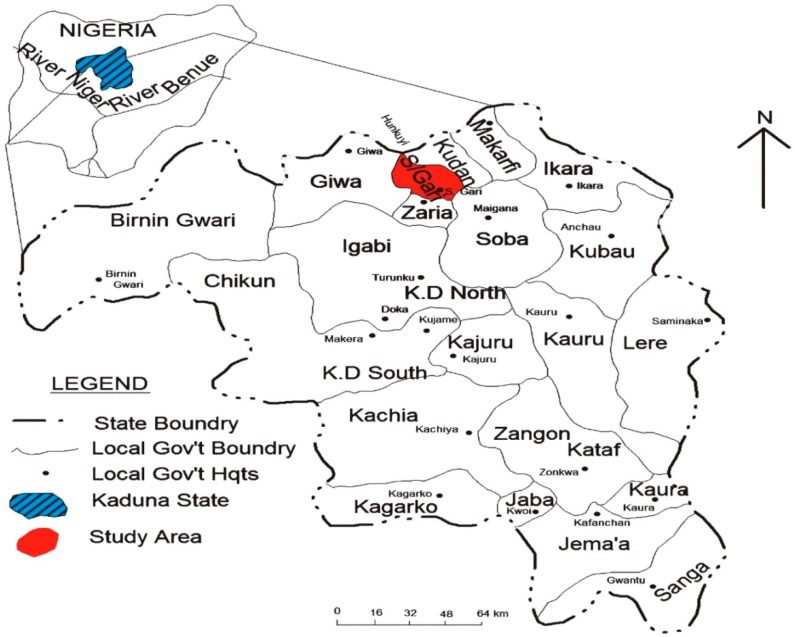
Map of Kaduna state of Nigeria showing the study area in Sabon/Gari Local Government.

The sampling has been carried out in the city of Zaria (11°3'N; 7°42'E), located in the North of Kaduna state, along the Kaduna-Kano highway [37]. Zaria comprises two Local Government Areas, namely Zaria and Sabon Gari, with the latter being the study area. Zaria urban area is composed of four districts, namely Zaria City, Tudun Wada, Sabon Gari and Samaru (Figure 1). The study area is characterised by a natural and stable ecosystem in the Northern Guinea Savannah zone, with a discontinuous layer of sparsely distributed short trees followed by relatively continuous layers of tall, medium and short grasses [37]. The dry season lasts from November to April and the wet one from May to October. The mean annual rainfall is about 1000 mm [38].

### 4.2. Sampling Strategy

Four different sampling points have been considered for the study: faecal samples from cattle at a large abattoir, effluents discharged from the abattoir itself, the water from the river Koreye collecting the effluents from the abattoir, and the vegetables grown at a nearby farm using the river water to irrigate crops.

A total of 200 faecal samples have been collected from cattle immediately before stunning. Faecal material has been taken inside and around the rectum of single animals. The abattoir has been visited once a week for a 20 weeks period and ten different animals were individually sampled during each visit.

The effluent from the abattoir has been sampled at three different points along its pathway to the river. The drainage system within the abattoir was the first sampling point (E-1), followed by a second one placed about 100 m away from the abattoir along the effluent’s way to the river Koreye (E-2) and at the junction where the effluent spills into the water stream (E-3). Each of these points has been sampled separately. Clean sterile bottles have been used to collect 100 mL of the effluent samples. Three samples have been taken at each sampling point once a week for a 20 weeks period for a total of 180 effluent samples.

The water from the river Koreye has been sampled in 100 mL aliquots using clean sterile bottles from the bank of the stream at the conjunction with the effluent (S-1), the middle of the stream (S-2) and at about 500 m downstream in proximity of a farm where vegetables were grown (S-3). One sample has been taken at each sampling point once a week for a 20 weeks period for a total of 60 water samples.

Most of the vegetables grown in the farm were lettuce, cabbage and carrot. Five samples of each of the types of vegetables have been collected as portions of 25 grams once a week for a 20 weeks period. As a whole, 300 samples have been collected and analysed.

### 4.3. Laboratory Procedure for Isolation and Identification of E. coli

One gr of each of the faecal samples has been suspended in 10 mL of Trypticase Soy Broth (TSB, Oxoid, Basingstoke Hampshire, UK) and incubated at 37 °C for 24 h. One hundred µL of enrichment broth have been streaked onto Eosin Methylene Blue agar (EMB, Oxoid) and Sorbitol MacConkey agar (SMAC, Oxoid) plates and incubated at 37 °C for 24 h. One ml of each effluent sample has been inoculated into 9 mL of TSB modified (Oxoid) supplemented with novobiocin 20 mg/L and incubated at 37 °C. After 24 h ten µL of the enrichment culture have been streaked on EMB agar plates and incubated at 37 °C for 24 h.

Membrane filtration technique has been used for the isolation and the identification of *E. coli* from water samples. Single sterile 0.45 µm pores filter disks (Pall Corporation) have been placed in a filtration unit to filter each water sample. The filter membranes have been placed on EMB agar plates and incubated at 37 °C for 24 h.

Each vegetable sample (25 grams) has been homogenized in 225 mL of buffered peptone water (BPW, Oxoid) and incubated at 37 °C for 12–18 h according to the method described by Jalil *et al.*, [39]. One hundred µL of the enrichment culture have been streaked on EMB agar for isolation of *E. coli*. Colonies showing a greenish metallic sheen with dark centres on EMB were considered as *E. coli* and further characterised. For each of the samples one colony resembling *E. coli* was picked up from the EMB plates and biochemically confirmed as previously described [40].

### 4.4. Screening for STEC Using a Commercial Enzyme Immunoassay (EIA)

All the confirmed *E. coli* were screened for the production of Shiga toxins by using the Ridascreen^®^Verotoxin (R-Biopharm, Darmstadt, Germany) according to the manufacturer’s instructions (http://www.r-biopharm.de/).

### 4.5. Characterization of E. coli Isolates by Vero Cell Assay and PCR

The characterization of the *E. coli* strains isolated during the study has been conducted at the European Reference Laboratory for *E. coli* in Rome, Italy. The *E. coli* isolates positive to the Ridascreen EIA have been subjected to the Vero cell assay to confirm the production of Shiga toxins. Each isolate has been streaked onto Mac Conkey agar plate and incubated at 37 °C for 18 h. A single colony of each strain has been inoculated into 1 mL of TSB and incubated at 37 °C for 18 h. Five hundred µL of each culture have been centrifuged at 13,000 rpm for 10 min. Twenty µL of the 0.22 μm-filtered supernatant have been inoculated onto Vero cell semi-confluent monolayers in microtitre plates and incubated for 37 °C in presence of 5% CO_2_ as previously described [41]. The presence of cytopathic effect was microscopically assessed after 24, 48 and 72 h from the inoculum.

Twenty-five µL of the TSB cultures were added to 975 µL of sterile water and boiled at 100 °C for 15 min for the following PCR analysis of the presence of genes associated with diarrheagenic *E. coli*. After a centrifugation at 13,000 ×*g* for 5 min, 5 µL of the supernatant have been used as a template.

All the oligonucleotides used in the present study have been listed in Table 3. In detail, a multiplex PCR has been carried out to confirm the presence of *stx*_1_, *stx*_2_ and *eaeA* genes of EHEC and EPEC as previously described [42]. A multiplex PCR has also been performed for the screening of the presence of the EAggEC-associated genes *aggR* and *aaiC* using the primers pairs described by Boisen *et al.*, [30], while the presence of the *aatA* gene has been investigated by PCR as described elsewhere [43]. PCR was also used for the detection of *ipaH* and the Subtilase cytotoxin (SubAB)-coding genes, associated respectively to EIEC and SubAB-producing *E. coli*, using the primers pairs and conditions described in the respective papers [44,45]. The amplification products have been analysed by electrophoresis on 1% or 2% agarose gel for single and multiplex PCR reactions, respectively. Finally, a real-time PCR has been used for the detection of *lt*, *sth* and *stp* enterotoxin-coding genes of ETEC as described by Liu and colleagues [46].

**Table 3:** Primers used for pathogenic *E. coli* characterisation.

Primer Name	Sequence (5'- 3' )	Amplicon Size (bp)	Reference
***stx*_1_F**	ATAAATCGCCATTCGTTGACTAC	180	[42]
***stx*_1_R**	AGAACGCCCACTGAGATCATC		
***stx*_2_F**	GGCACTGTCTGAAACTGCTCC	255	[42]
***stx*_2_R**	TCGCCAGTTATCTGACATTCTG		
***eaeA*F**	GACCCGGACAAGCATAAGC	384	[42]
***eaeA*R**	CCACCTGCAGCAACAAGAGG		
***aggR*F**	GCAATCAGATTAARCAGCGATACA	426	[30]
***aggR*R**	CATTCTTGATTGCATAAGGATCTGG		
***aaiC*F**	TGGTGACTACTTTGATGGACATTGT	313	[30]
***aaiC*R**	GACACTCTCTTCTGGGGTAAACGA		
**pCVD432/start (aat)**	CTGGCGAAAGACTGTATCAT	630	[43]
**pCVD432/stop (aat)**	CAATGTATAGAAATCGCCTGTT		
***Shig-1***	TGGAAAAACTCAGTGCCTCT	423	[45]
***Shig-2***	CCAGTCCGTAAATTCATTCT		
***RTSubAB*F**	GCAGATAAATACCCTTCACTTG	232	[44]
***RTSubAB*R**	ATCACCAGTCCACTCAGCC		

## 5. Conclusions

The results from this study provide further evidence that pathogenic *E. coli* of zoonotic origin can contaminate the environment as a result of the discharge of untreated abattoir effluent. Additionally, the lack of enforcement of good hygiene practices may ease the release and persistence of multiple pathogenic *E. coli* strains in the abattoir environment, including those belonging to human-borne pathogroups, making such a setting a unique favourable environment for bacteria to bacteria interaction and exchange of genetic material possibly leading to the emergence of new pathogenic strains with shuffled virulence features. The isolation of *E. coli* strains with similar virulence genes assets from either the slaughtered animals or the abattoir effluent and the vegetables grown at a farm close to the slaughterhouse suggests that the water from the river collecting the abattoir effluents and used to irrigate the crops may have served as a source of pathogenic *E. coli* for the contamination of vegetables.
